# Comparison of Bone Regeneration between Porcine-Derived and Bovine-Derived Xenografts in Rat Calvarial Defects: A Non-Inferiority Study

**DOI:** 10.3390/ma12203412

**Published:** 2019-10-18

**Authors:** Eun-Bin Bae, Ha-Jin Kim, Jong-Ju Ahn, Hyun-Young Bae, Hyung-Joon Kim, Jung-Bo Huh

**Affiliations:** 1 Department of Prosthodontics, Dental Research Institute, Dental and Life Science Institute, BK21 PLUS Project, School of Dentistry, Pusan National University, Yangsan 50612, Korea; 0228dmqls@hanmail.net (E.-B.B.); tarov0414@daum.net (J.-J.A.); h.02@hanmail.net (H.-Y.B.); 2 Department of Oral Physiology, Dental Research Institute, Dental and Life Science Institute, School of Dentistry, Pusan National University, Yangsan 50612, Korea; ya120010@naver.com

**Keywords:** bone regeneration, bone substitute, xenograft, porcine bone

## Abstract

The present study aimed to compare the bone-regeneration capacity of porcine-derived xenografts to bovine-derived xenografts in the rat calvarial defect model. The observation of surface morphology and in vitro cell studies were conducted prior to the animal study. Defects with a diameter of 8 mm were created in calvaria of 20 rats. The rats were randomly treated with porcine-derived (Bone-XP group) or bovine-derived xenografts (Bio-Oss group) and sacrificed at 4 and 8 weeks after surgery. The new bone regeneration was evaluated by micro-computed tomography (μCT) and histomorphometric analyses. In the cell study, the extracts of Bone-XP and Bio-Oss showed a positive effect on the regulation of osteogenic differentiation of human mesenchymal stem cells (hMSCs) without cytotoxicity. The new bone volume of Bone-XP (17.52 ± 3.78% at 4 weeks and 32.09 ± 3.51% at 8 weeks) was similar to that of Bio-Oss (11.6 ± 3.88% at 4 weeks and 25.89 ± 7.43% at 8 weeks) (*p* > 0.05). In the results of new bone area, there was no significant difference between Bone-XP (9.08 ± 5.47% at 4 weeks and 25.22 ± 13.56% at 8 weeks) and Bio-Oss groups (5.83 ± 2.56% at 4 weeks and 21.68 ± 11.11% at 8 weeks) (*p* > 0.05). It can be concluded that the porcine-derived bone substitute may offer a favorable cell response and bone regeneration similar to those of commercial bovine bone mineral.

## 1. Introduction

An insufficient alveolar bone volume can produce the problems of implant insertion and prognosis, therefore, bone graft materials have been commonly used to reconstruct the osseous defects in the implant and periodontic surgeries. Bone grafting not only fills the boneless space but also provides structural stability and facilitates bone tissue growth [1]. For successful bone regeneration, numerous bone substitutes have been developed and introduced using autogenous bone, allografts, synthetic bone, and xenografts [2,3].

Among them, autogenous bone has been considered a gold standard for bone regeneration from biological and histological vantage points [4]. Nonetheless, autografts have not frequently been applied in clinical application due to concerns about additional injury, donor site limitations, and morbidity from the bone harvest [5,6]. On the contrary, xenogeneic bone substitutes derived from bones of other species also a have sufficient osteoconductivity and biocompatibility and have generally been used in the dental field [7]. Besides, unlike autogenous bone, xenografts circumvent a second operative site and have no limit in terms of the available bone amount [1,8].

Currently, the deproteinized cancellous bovine bone matrix (DBBM) is mainly transplanted in alveolar bone defects and in sinus floor augmentation [9,10]. The most well-known DBBM in dentistry is Bio-Oss^®^ with successful preclinical and clinical results, which has similar porous structure to human bone and shows high biocompatibility with oral hard tissues, and also meets the criteria of bone conductivity [1,11,12,13]. Such a graft consists of hydroxyapatite prepared by alkaline and heat treatment (300 °C) for eliminating the organics of the bone. However, in using the bovine-derived bone substitute, the fear of bovine spongiform encephalopathy (BSE) transmission is still potentially inherent [14]. BSE is a kind of transmissible spongiform encephalopathies (TSE), a fatal neurodegenerative disorder that can be transmitted to humans [15].

Porcine bone also shares a similar physiological, anatomic, and genetic makeup to human [16,17,18]. Hence the porcine-derived xenografts with relatively low zoonosis risk have been recently developed and made commercially available as an alternative to bovine-derived xenografts [16,19,20]. Porcine xenogeneic bone substitute has crystal structures similar to the human-derived bones like bovine bone [18,21]. Moreover, the stiffness and Ca/P ratio of porcine xenograft are closer to human trabecular bone than bovine bone [22]. Despite the porcine-derived xenografts having crystalline structures resembling human osseous tissue seem to be a profitable bone graft, the researches and data on porcine-derived xenografts still lack compared to bovine-derived grafts.

In the present study, we expected that the porcine-derived xenograft (Bone-XP^®^) recently introduced in the dental field may serve either non-inferior or superior effects on bone regeneration compare to the well-known commercially available bovine-derived bone substitute (Bio-Oss^®^). Thus, this study was undertaken to compare the bone regeneration capacity of porcine-derived xenografts to bovine-derived xenografts in the rat calvarial defect model.

## 2. Materials and Methods

### 2.1. Experimental Xenogenetic Bone Substitues

Two kinds of commercially available xenogeneic bone substitutes derived from different species (porcine and bovine) were used in this study. For porcine-derived xenograft (Bone-XP group), Bone-XP^®^ (particle size of 0.2 mm–1.0 mm) was purchased from Medpark (Busan, Korea). Bovine bone graft material (Bio-Oss group), Bio-Oss^®^ (particle size of 0.25–1.0 mm), was purchased from Geistlich Biomaterials (Wolhusen, Switzerland).

### 2.2. In Vitro Study

#### 2.2.1. Scanning Electron Microscopy (SEM)

To compare the surface morphologies of porcine and bovine xenogeneic bone substitute, scanning electron microscopy (SEM) observation was conducted. After coating the Bio-Oss and Bone-XP with Au using a sputter coater (SCD 005, BAL-TEC, Balzers, Liechtenstein), the SEM (Hitachi S3500N, Hitachi, Tokyo, Japan) was operated at 15 kV.

#### 2.2.2. Preparation of Extracts

We mixed 1 g of each xenogeneic bone substitute (Bio-Oss and Bone-XP) with 10 mL of alpha-modification of Eagle’s medium (α-MEM; Welgene, Deagu, Korea) and stored at 37 °C and 5% CO_2_ for 1 day. Each suspension was centrifuged once, for 5 min at 1200× *g*. These suspensions were filtered through the membrane (pore size: 0.2 μm) and stored 4 °C before use. The concentration of extract solutions in culture media ranging 20% was treated.

#### 2.2.3. Culture of Human Bone Marrow Mesenchymal Stem Cells (hMSCs)

Human mesenchymal stem cells (hMSCs) (LONZA, Walkersville, MD, USA) were used for this in vitro cell study. hMSCs culture medium consisted of alpha-modification of Eagle’s medium (α-MEM; Welgene Inc., Deagu, Korea) supplemented with 10% fetal bovine serum (FBS; Gibco BRL, Carlsbad, CA, USA), 100 U/mL penicillin and 100 μg/mL streptomycin (Gibco BRL), at 37 °C, 5% CO_2_. The cultured medium was changed regularly every three days. The cells were detached with 0.25% trypsin/1 mM EDTA (Gibco BRL) and passaged after reaching 80%–90% confluence. hMSCs between Passages 4 and 5 were used for all the experiments.

#### 2.2.4. Differentiation toward Osteoblasts

For osteogenic differentiation, hMSCs were seeded into a 48-well plate (3 × 10⁴ cells/mL) in α-MEM and incubated at 37 °C and 5% CO_2_ for 24 h. The media was replaced to osteogenic differentiation media, i.e., 10% α-MEM supplemented with 50 μg/mL ascorbic acid-2-phosphate (Sigma-Aldrich, Milan, Italy) and 10 mM β-glycerophosphate (Sigma-Aldrich), which function as a positive control. Media was changed every two days.

#### 2.2.5. Cell Viability and Proliferation Assay

The cell viability and proliferation were assessed using the CCK-8 assay kit (Dojindo, Rockville, MD, USA) at 0, 1, 2, and 3 days. hMSCs were seeded into 48-well plates (Nunc, Roskilde, Denmark) at a density of 1 × 10^4^ cells/mL. Each plate was pre-incubated in a humidified incubator with 5% CO_2_ at 37 °C, and 20 μL of the CCK-8 solution was added to each well and then incubated for 2 h. 100 μL/well aliquots were transferred to a 96-well plate (Nunc) and absorbance was measured at 450 nm using an Opsys MR micro-plate reader (DYNEX Technologies Inc., Denkendorf, Germany). The cell viability was determined in 24 h absorbance data. The cell proliferation rate was assessed in optical density (OD) units.

#### 2.2.6. Alkaline Phosphatase (ALP) Activity Assay

To evaluate the osteogenic differentiation, we used the Leukocyte Alkaline Phosphatase Kit (Sigma-Aldrich) according to the manufacturer’s protocol. hMSCs were plated into 48-well plates (Nunc) at a density of 3 × 10^4^ cells/mL and cultured for 3 and 8 days in the osteogenic media. Quantification of the staining images were done using ImageJ software program (U.S. National Institutes of Health, Bethesda, MD, USA).

#### 2.2.7. Real-Time Polymerase Chain Reaction (PCR) Analysis

The real-time polymerase chain reaction (PCR) analysis was performed to examine the gene level of osteogenic differentiation markers. hMSCs were seeded into a 6-well plate (2 × 10⁴ cells/mL). The cells cultured under the basal α-MEM, and the osteogenic α-MEM media were used as controls. Five days after the induction of osteogenesis, total RNA was extracted from different treated cells by using TRIzol (Life Technologies, Grand Island, NY, USA), and the total RNA concentration was measured by using a NanoDrop ND-1000 spectrophotometer (Technologies Inc., Wilmington, DE, USA). Complementary DNA (cDNA) was synthesized from 1.5 μg of the RNA. Amplification was done by using SYBR Green Master Mix reagents (Kapa Biosystems, Woburn, MA, USA) and an ABI 7500 instrument (Applied Biosystems, Carlsbad, CA, USA) according to the manufacturer’s protocols. The relative gene expressions (*ALP*, Osteopontin (*OPN*), and Runt-related transcription factor 2 (*RUNX2*)) were calculated using the relative 2-ΔΔCt method. The control gene, *Actin*, was used to normalize the target genes. All reactions were performed in three samples. The primers were synthesized and provided by Bionics (Daejeon, Korea) (Table 1).

### 2.3. In Vivo Animal Study

#### 2.3.1. Experimental Animal and Operative Procedures

**Figure 1:**
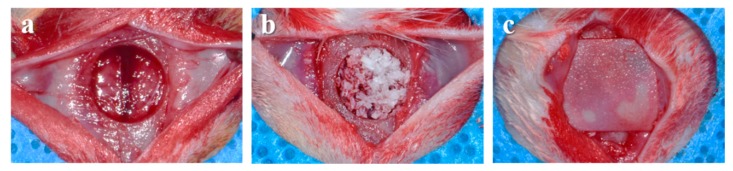
Operative procedures using rat calvarial defect model. (**a**) Created calvarial defect, (**b**) insertion of bone grafts, (**c**) placement of collagen membrane.

Twenty Sprague-Dawley rats (male, 13-week-old, Koatech, Pyeongtaek, Korea) were used in this animal experiment. The individually caged rats were fed with rodent pellets and water and adapted to the laboratory for a week prior to surgery. The room conditions were kept constant at a temperature of 25 ± 1 °C and at a humidity of 55 ± 7%. All the animal care and surgical procedures were done at the Laboratory Animal Resource Center of Pusan National University and were approved by the Institutional Animal Care and Use Committee of Pusan National University (PNU-2018-2101). During the surgical procedure, the rats were anesthetized using intramuscular injection of a combination of xylazine (Rompun, Bayer Korea, Seoul, Korea) and tiletamine zolazepam (Zoletil50, Virbac Korea, Seoul, Korea) for general anesthesia. The surgical sites of rats were shaved and then disinfected with povidone-iodine (Betadine, Korea Pharma, Seoul, Korea). The local anesthesia was performed using 2% lidocaine (Huons, Soengnam, Korea). After making a sagittal incision across the center of the skull using surgical scalpel blade (No. 15, Swann-Morton Ltd., Sheffield, UK), the cranium was exposed by raising the full thickness flap. Using a saline-cooled trephine bur (Osung, Kimpo, Korea), a critical-sized osseous defect (8 mm in diameter) was made in the center of the skull (Figure 1a). The quantified xenogeneic bone grafts (0.03 ± 0.002 g) were randomly implanted in each defect (Figure 1b). The defect was covered with a resorbable collagen membrane (10 × 10 mm, Cola-D, Medpark, Seoul, Korea) (Figure 1c). Skin and periosteum were sutured using 4-0 Vicryl suture (Ethicon, Livingston, UK). At 4 and 8 weeks after surgery, the experimental rats were sacrificed by CO_2_ inhalation. The tissue samples were carefully harvested and immersed in 10% neutral buffered formalin (Sigma-Aldrich) for 2 weeks.

#### 2.3.2. Micro-Computed Tomography (μCT) Analysis

**Figure 2:**
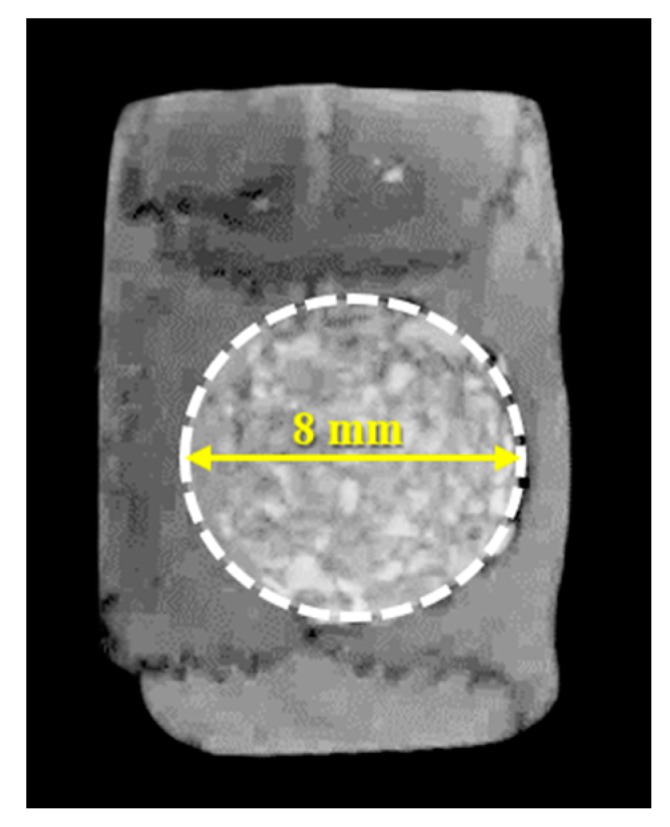
Area of interest (AOI) for volumetric analysis.

All the harvested samples were scanned using μCT (SMX-90CT, Shimadzu, Kyoto, Japan) at 90 kV, intensity of 109 μA for measuring the percentage of new bone volume. The new bone volume was calculated using a customized program corded by cording software (MATLAB 2018b, MathWorks, Natick, MA, USA). The area of interest (AOI) was set as the same for all the specimens (diameter of 8 mm, height of 1.5 mm) (Figure 2). The images were divided into soft tissue, bone graft material and new bone and were classified by threshold values.

#### 2.3.3. Histological and Histomorphometric Analysis

**Figure 3:**
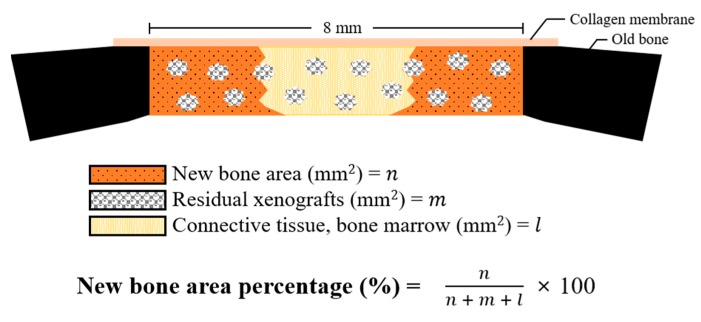
Schematic diagram of histometric analysis.

To prepare the decalcified histological sections, fixed-tissue specimens were soaked in 2.5% sodium hypochlorite/17% Ethylenediaminetetraacetic acid (EDTA) solution, and the solution was replaced daily for 2 weeks. Afterwards, specimens were dehydrated in a graded ethanol series and xylene and were embedded in paraffin. The paraffin blocks were cut into 3–4 µm thickness using a microtome (Microm HM 325, Waltham, MA, USA) and then attached to poly-L-lysine-coated slides. The tissue specimens were stained with hematoxylin-eosin (H&E) and Masson’s trichrome (MT) staining solutions and photographed by optical microscope (Olympus BX, Tokyo, Japan) attached CCD camera (Polaroid DMC2 digital Microscope Camera, Polaroid, Cambridge, MA, USA). The newly formed bone area at the defect sites were consistently measured using a i-solution (IMT, Daejeon, Korea) by a single investigator (Figure 3).

### 2.4. Statistical Analysis

All the statistical analyses were performed using SPSS statistical analysis software (ver. 24.0, IBM, Armonk, NY, USA) with a confidence level of 95% (*p* < 0.05). The in vitro and in vivo results were analyzed by using the independent student’s *t*-test to determine the significance of differences between groups.

## 3. Results

### 3.1. In Vitro Findings

#### 3.1.1. Observations of Surface Morphology

**Figure 4:**
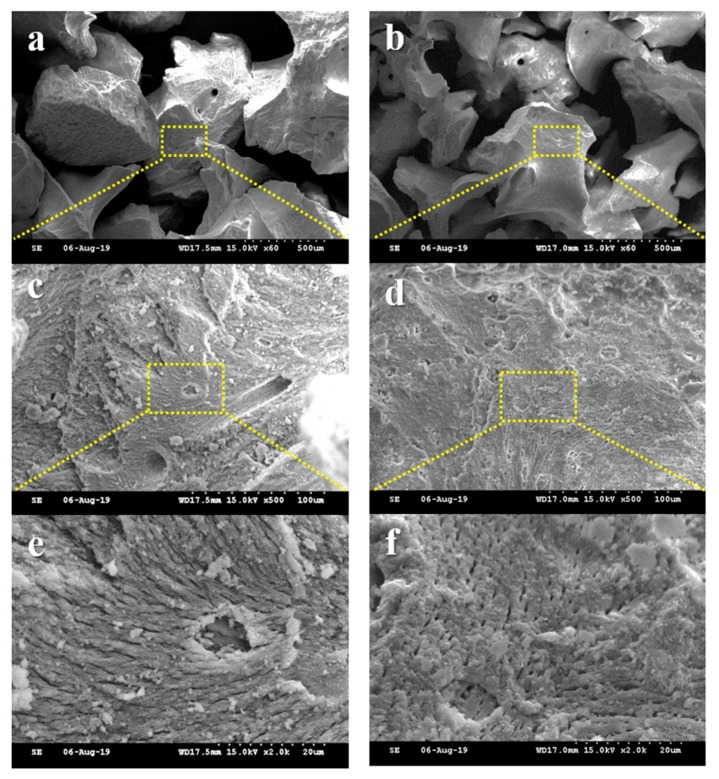
Comparative scanning electron microscope (SEM) images of each group. (**a**,**c**,**e**) Bio-Oss and (**b**,**d**,**f**) Bone-XP groups. [Original magnification: ×60 (**a**,**b**), ×500 (**c**,**d**), ×2000 (**e**,**f**)].

To investigate the surface morphologies of bone grafts, the SEM images were captured at magnification of ×60, ×500, and ×2000 (Figure 4). The xenografts of both groups showed similar particle size (Figure 4a,b) and macro-porous structure (Figure 4c–f) exhibiting the rough surfaces.

#### 3.1.2. Cell Viability and Proliferation

**Figure 5:**
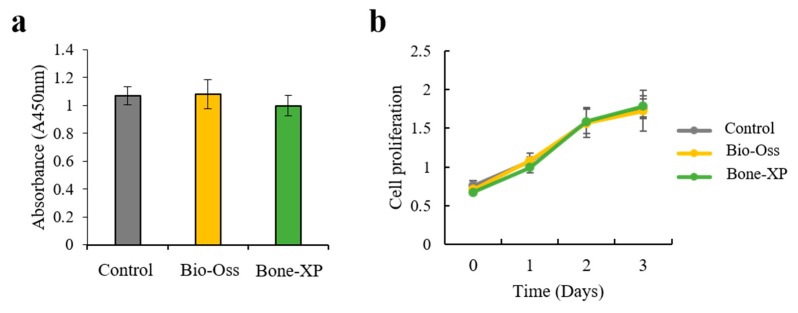
(**a**) Cell viability and (**b**) proliferation of extracts on human mesenchymal stem cells (hMSCs).

The viability and proliferation of hMSCs on the two different extracts were analyzed using the CCK-8 assay, 0, 1, 2, and 3 days after cell seeding. At each time point, cell numbers were calculated following the normalization of the absorbance units to that obtained for the cells cultured on the Control group (Figure 5). At 1 day, the number of viable cells was similar for the extracts of Bio-Oss group and Bone-XP group and no significant differences were observed (Figure 5a). After 1, 2, and 3 days of culture, the cell proliferation of hMSCs on the two extracts was no different to that of the Control group (Figure 5b). These results indicate that the hMSCs cultured on two extractsf had no affect toxicity, and the proliferation rate was quite modest.

#### 3.1.3. Alkaline Phosphatase (ALP) Staining

**Figure 6:**
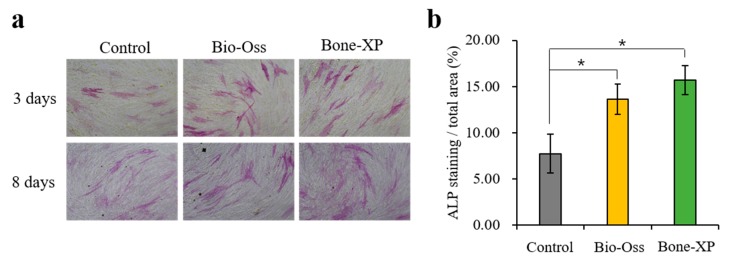
Cell osteogenic differentiation assay. (**a**) Alkaline phosphatase (ALP) staining and (**b**) the quantitative analysis. The symbol * indicates statistical significance compare to control (* *p* < 0.05).

The alkaline phosphatase staining was conducted to analyze the effect of Control untreated hMSCs and those treated either with Bio-Oss group or Bone-XP group extracts after incubation for 3 and 8 days (Figure 6). On day 3, ALP staining slightly increased in the cells cultured on both the Bio-Oss group and Bone-XP group compared to the Control group. In addition, there was no obvious staining difference between Bio-Oss group and Bone-XP group-treated hMSCs. On day 8, both Bio-Oss group and Bone-XP group treated hMSCs, which were not substantially different from each other, showed higher staining than the Control, untreated cells. These data suggest that the extracts of Bio-Oss and Bone-XP had a positive effect on the regulation of osteogenic differentiation of hMSCs.

#### 3.1.4. Analysis of Real-time Polymerase Chain Reaction (PCR)

**Figure 7:**
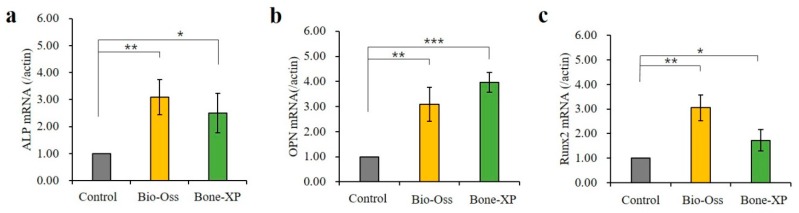
Real-time polymerase chain reaction (PCR) analysis of hMSCs on extracts. (**a**) *ALP*, (**b**) *OPN,* and (**c**) *RUNX2* were selected as the osteogenic differentiation related genes. The symbol * indicates statistical significance compare to control group (* *p* < 0.05, ** *p* < 0.01, *** *p* < 0.001).

It is important to note that *ALP* and *RUNX2* were an early marker of osteoblastic lineage, and to evaluate the effects of Bio-Oss and Bone-XP extracts on osteoblast differentiation. The levels of osteogenesis-related genes were examined by real-time PCR (Figure 7). hMSCs were cultured in medium supplemented with osteogenic factors and 20% extracts for 5 days. Compared with control, untreated hMSCs, the up-regulated genes by Bio-Oss and Bone-XP extracts induced the factors involved in osteoblast differentiation and matrix mineralization, *ALP* and *OPN* (Figure 7a,b) and key osteogenic transcriptional factor; *RUNX2* (Figure 7c). Additionally, there was no statistically significant difference between Bio-Oss and Bone-XP-treated hMSCs (Figure 7a,b).

### 3.2. In Vivo Findings

#### 3.2.1. Clinical Findings

All the rats survived during the operative procedures and healing periods. In all the surgical sites, any side effects such as inflammation, swelling, or exposure of the specimens were not observed.

#### 3.2.2. Volumetric Findings

**Figure 8:**
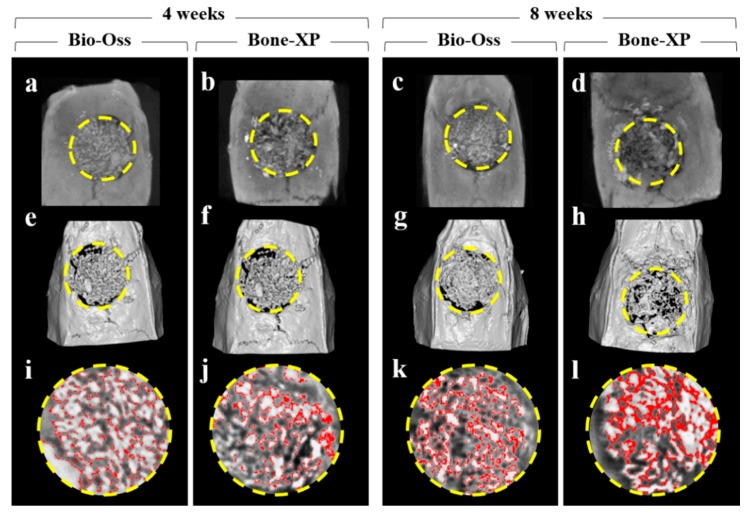
Micro-computed tomography (μCT) analysis images. (**a**–**d**) μCT images. (**e**–**h**) 3D reconstructed μCT images. (**i**–**l**) Classified new bone in area of interest (AOI). Yellow circle: AOI, Red colored area: newly formed bone.

**Figure 9:**
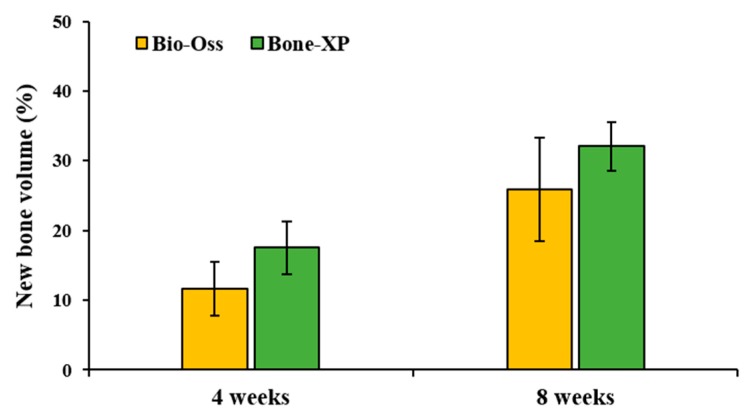
New bone volume percentages within area of interest (AOI).

On μCT images, bone grafts were well-positioned in the AOI without scattering of particles (Figure 8a–d). Within the AOI, the similar amount of new bone areas classified by threshold were observed in both experimental groups at 4 weeks and 8 weeks, respectively (Figure 8i–l). At 4 weeks, the new bone volumes (%) of Bio-Oss and Bone-XP groups were 11.6 ± 3.88% and 17.52 ± 3.78%, respectively (Table 2 and Figure 9). At 8 weeks, new bone volumes of Bio-Oss group and Bone-XP group were 25.89 ± 7.43% and 32.09 ± 3.51%, respectively. There was no significant difference in new bone volume between groups at both 4 and 8 weeks (*p* > 0.05).

#### 3.2.3. Histological and Histomorphometric Findings

**Figure 10:**
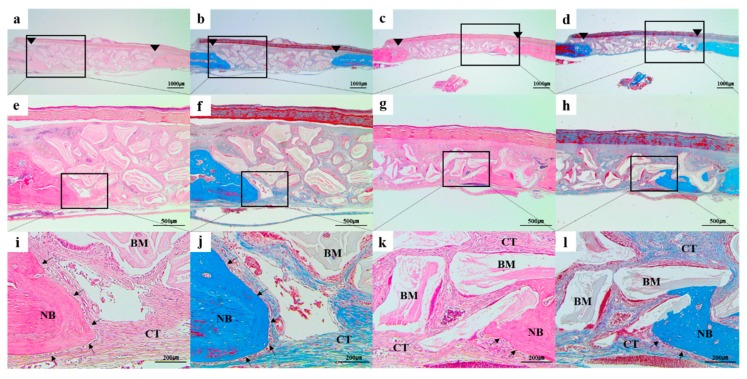
Histologic sections of (**a**,**b**,**e**,**f**,**i**,**j**) Bio-Oss and (**c**,**d**,**g**,**h**,**k**,**l**) Bone-XP groups at 4 weeks post-surgery. (**a**,**c**,**e**,**g**,**i**,**k**) haematoxylin and eosin (H&E) stained slides; (**b**,**d**,**f**,**h**,**j**,**l**) Masson’s trichrome (MT) stained slides; Arrowhead: original defect edge; Arrow: lines of osteoblasts NB: newly generated bone; CT: connective tissue; BM: residual bone grafts. [Original magnification: (**a**–**d**) ×12.5, (**e**–**h**) ×40, (**i**–**l**) ×100].

**Figure 11:**
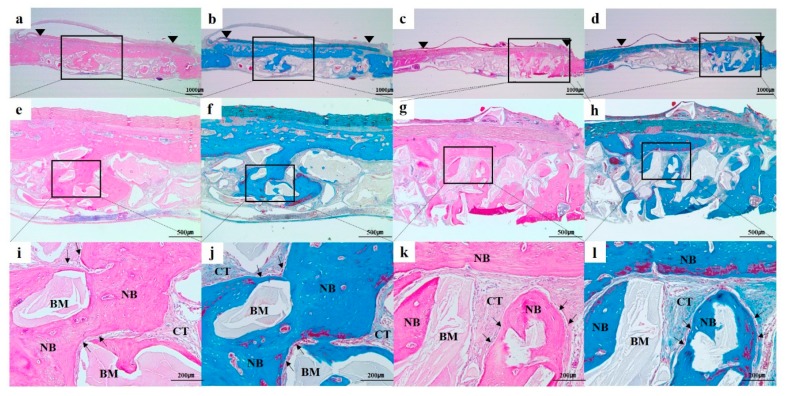
Histologic sections of (**a**,**b**,**e**,**f**,**i**,**j**) Bio-Oss and (**c**,**d**,**g**,**h**,**k**,**l**) Bone-XP groups at 8 weeks post-surgery. (**a**,**c**,**e**,**g**,**i**,**k**) haematoxylin and eosin (H&E) stained slides; (**b**,**d**,**f**,**h**,**j**,**l**) Masson’s trichrome (MT) stained slides; Arrowhead: original defect edge; Arrow: lines of osteoblasts NB: newly generated bone; CT: connective tissue; BM: residual bone grafts. [Original magnification: (**a**–**d**) ×12.5, (**e**–**h**) ×40, (**i**–**l**) ×100].

In all the histologic sections, the implanted bone-graft materials were holding a stable position within the defective site and showed good space-maintenance for bone reconstruction (Figure 10 and Figure 11). At all the time point of 4 and 8 weeks, there were no remarkable inflammatory reactions such as presence of inflammatory cells or hematoma forms, and a number of active osteoblasts’ lines could be easily found surrounding the newly formed bone (Figure 10i–l and Figure 11i–l). In addition, connective tissues and bone marrow were observed around the residual bone graft materials. At 4 weeks after surgery, the new bones were minimally regenerated from the defect margin in both groups (Figure 4). The bone rebuilding was time-dependent, the mineralization and amount of new bone were increased at 8 weeks compared to those of 4 weeks in both groups (Figure 11).

**Figure 12:**
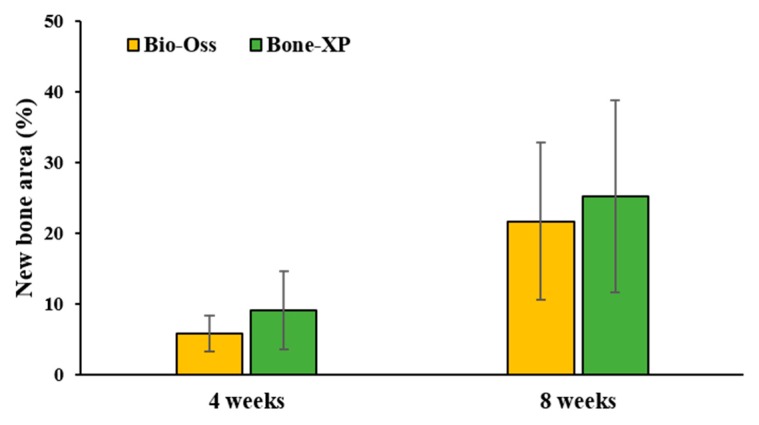
New bone area percentages within area of interest (AOI).

The histomeric results of new bone area at 4 and 8 weeks after surgery are shown in Table 3 and Figure 12. At 4 weeks post-surgery, the mean ± standard deviation (SD) of new bone areas (%) in Bio-Oss and Bone XP groups were 5.83 ± 2.56%, 9.08 ± 5.47%, respectively. At 8 weeks, the new bone areas of Bio-Oss and Bone XP groups were 21.68 ± 11.11% and 25.22 ± 13.56%, respectively. There was no significant difference in new bone volume between groups at both 4 and 8 weeks (*p* > 0.05).

## 4. Discussion

Porcine-derived xenografts are a biocompatible material and have similar structures to that of human bone [23]. Bone-XP^®^ used in our study is a heat-treated mineralized porcine bone, commercially available in dentistry. To remove the residual organic components, Bone-XP^®^ was produced by thermal-treatment at high temperature. The high-temperature thermal treatment gives a higher crystalline structure and longer hydroxyapatite crystal [24,25]. While the heat-treatment may influence the Ca/P ratio, it is reported that the Ca/P ratio of Bone-XP (1.65–1.66) is closer to the Ca/P ratio of human bone (1.68–1.71) than that of bovine bone (1.92) [22]. An et al. [26] conducted animal experiments using the porcine-derived xenogeneic bone substitutes and reported the uniform new bone formation in rabbit tibia at 16 weeks post-surgery. They also conducted the multicenter clinical trial in orthopedics and proved the safety, stability, and appropriate absorption rate of porcine bone in the ilium. In addition, in the case report for alveolar ridge preservation using porcine bone mineral, the smooth new bone formation could be observed around the grafted xenografts without any adverse events such as inflammatory reaction or fibrous film formation [27]. The measured new bone rate (37.4%) was not inferior to the similar previous studies on ridge preservation using allogeneic bone and DBBM [27,28,29]. Even though there are lots of advantageous points of thermal-treated mineralized porcine xenografts, since most of the previous works on porcine bone grafts have been as to the collagenized porcine xenografts (CPX) [30,31,32,33], it is a lack of relevant research on anorganic porcine xenograft materials compared to CPX or DBBM. To expand the choice of bone grafts in dental applications, therefore, we conducted the in vitro and in vivo evaluation of porcine-derived xenografts in comparison with bovine-derived xenografts.

In our SEM observation, both Bone-XP and Bio-Oss groups exhibited similar naturally rough surfaces. The surface roughness of material influences cell morphology, behavior, and adhesion [34]. Hatano et al. [35] reported that osteogenic cells grown on the micro-rough surface showed higher proliferation, ALP activity, and osteogenic gene expression in comparison with cells on the smooth surface. Therefore, the micro-rough surfaces of Bone-XP and Bio-Oss are considered to be favorable to adherence and proliferation of osteogenic cells.

MSCs can differentiate into cells of the mesodermal lineage, such as adipocyte, osteocyte, and chondrocyte, etc. The proliferative and osteogenic characteristics of MSCs are used for reconstructing bone defects in clinical conditions [36,37]. Previous studies demonstrated that Bio-Oss has a positive effect on the osteogenic ability of the hMSCs [38]. In this study, hMSCs were used to evaluate the osteogenic activity of Bone-XP in comparison with the Bio-Oss. The cells cultured on the two different extracts of bone grafts showed no significant effect in terms of their cytotoxicity and proliferation rate. Upon osteogenic differentiation, both investigated extracts showed significant increases compared to control untreated cells. To determine the mechanism of osteogenic induction by the investigated extracts, we examined the related gene expressions by quantitative PCR (qPCR). In bone, *ALP* is localized on the entire cell surface of pre-osteoblasts and has long been used as an osteoblastic marker [39,40]. *OPN*, a secreted matrix glycoprotein, is biosynthesized by preosteoblast, osteoblast, osteocytes and plays a significant role in the bone remodeling procedure [41,42]. *RUNX2*, a key transcription modulator, is the main transcription factor for osteoblast differentiation and bone formation. In our study, both extracts of experimental xenografts induced ALP, OPN, and RUNX2 expressions in comparison to the control, but there was no significant difference between investigated extracts. The results of osteogenic differentiation and gene expression suggest that the Bone-XP is an osteoinductive material and can be used as a replacement for the Bio-Oss.

From a surgical standpoint of our in vivo experiment, Bone-XP gave favorable handling properties similar to Bio-Oss and could be easily implanted into the bone defects. In the study on the physicochemical property of porcine-derived bone which is essentially identical grafts with Bone-XP [1], the porcine-derived xenografts showed higher wettability than that of bovine-derived xenografts (Bio-Oss). High wettability of biomaterial can beneficially effect protein adsorption and cellular behavior [1,43]. In our surgical procedure, Bone-XP was easily wetted by body fluid after transplantation as well and it can be considered that Bone-XP is a relatively hydrophilic bone graft material.

This study conducted μCT, histologic, and histometric evaluations to compare the new bone formation between the porcine-derived and bovine-derived xenografts in a critical-sized rat calvarial defect. Osteoblast from the defect margin utilizes the grafted bone substitute as a frame upon which to regenerate bone [44]. The newly formed bone should replace bone grafts as the latter resorbs during the healing period. In our histological analysis, the lines of osteoblast were easily observed surrounding the grafted xenografts in all groups, and this indicates that both xenografts used in this study have an excellent osteoconductivity [45]. Furthermore, in the result of the volumetric and histometric analysis, Bone-XP and Bio-Oss group induced comparable proportions of new bone regeneration at all the time points of 4 and 8 weeks. These results demonstrated that Bone-XP has the non-inferior capacity of new bone regeneration compared to that of Bio-Oss.

Based on the results obtained in the present study, Bone-XP^®^, recently commercially available porcine xenograft, is a biocompatible, osteoinductive, and osteoconductive bone substitute in comparison with Bio-Oss^®^. However, this in vivo study was conducted using limited sample size and observation time points, and therefore future preclinical trials using large animal and clinical trials are needed to verify these results.

## 5. Conclusions

Within the limitations of the present study, it can be concluded that the newly investigated porcine-derived bone substitute, Bone-XP^®^, may offer a favorable cell response and bone regeneration similar to those of commercial bovine bone mineral.

## Figures and Tables

**Table 1:** Primer sequences used for real-time polymerase chain reaction (PCR) analysis.

Target Genes	Sequences
*ALP*	F: 5′-ATTTCTCTTGGGCAGGCAGAGAGT-3′
	R: 5′-ATCCAGAATGTTCCACGGAGGCTT-3′
*OPN*	F: 5′-AGACACATATGATGGCCGAGG-3′
	R: 5′-GGCCTTGTATGCACCATTCAA-3′
*Runx2*	F: 5′-CTCTACTATGGCACTTCGTCAGG-3′
	R: 5′-GCTTCCATCAGCGTCAACAC-3′
*Actin*	F: 5′-ACTCTTCCAGCCTTCCTTCC-3′
	R: 5′-TGTTGGCGTACAGGTCTTTG-3′

**Table 2:** New bone volume within area of interest (AOI). (n = 5).

		Groups	Mean	SD	*p*-Value
New bone volume (%)	4 weeks	Bio-Oss	11.6	3.88	0.092
		Bone-XP	17.52	3.78	
	8 weeks	Bio-Oss	25.89	7.43	0.38
		Bone-XP	32.09	3.51	

**Table 3:** New bone area within area of interest (AOI). (n = 5).

		Groups	Mean	SD	*p*-Value
New bone area (%)	4 weeks	Bio-Oss	5.83	2.56	0.139
		Bone-XP	9.08	5.47	
	8 weeks	Bio-Oss	21.68	11.11	0.273
		Bone-XP	25.22	13.56	
